# Development and performance evaluation of a qPCR-based assay for the fully automated detection of group B *Streptococcus* (GBS) on the Panther Fusion Open Access system

**DOI:** 10.1128/spectrum.00057-24

**Published:** 2024-04-29

**Authors:** Andy Caballero Méndez, Roberto A. Reynoso de la Rosa, Miguel E. Abreu Bencosme, Mayeline N. Sosa Ortiz, Eliezel Pichardo Beltré, Darah M. de la Cruz García, Nelson J. Piñero Santana, Joana C. Bacalhau de León

**Affiliations:** 1 Molecular Biology Department, Referencia Laboratorio Clínico, Santo Domingo Oeste, Santo Domingo, Dominican Republic; 2 Microbiology Department, Referencia Laboratorio Clínico, Santo Domingo Oeste, Santo Domingo, Dominican Republic; University of Maryland School of Medicine, Baltimore, Maryland, USA

**Keywords:** molecular diagnostic, NAAT, qPCR, group B *Streptococcus*, early-onset disease, laboratory-developed test (LDT)

## Abstract

*Streptococcus agalactiae* [group B *Streptococcus* (GBS)] poses a major threat as the primary cause of early-onset neonatal invasive disease, particularly when mothers are colonized rectovaginally. Although culture remains the gold standard for antepartum GBS screening, quantitative polymerase chain reaction (qPCR) offers advantages in terms of sensitivity and turnaround time. The aim of this study was to validate the clinical utility of an automated qPCR laboratory-developed test (LDT) for antepartum GBS screening using the Panther Fusion Open Access system (Hologic, California, USA). The LDT targeted a conserved region of the GBS *surface immunogenic protein* gene, demonstrating no cross-reactivity and high coverage (99.82%–99.99%). The limit of detection (LoD) was 118 CFU/mL. Comparison with commercial qPCR assays (Panther Fusion GBS and VIASURE *Streptococcus* B Real-Time) revealed an overall agreement of 99.7%, with a robust Cohen’s kappa coefficient of 0.992. Testing of 285 rectovaginal swabs from pregnant women and 15 external quality assessment samples demonstrated exceptional diagnostic performance of the LDT, achieving a diagnostic sensitivity and specificity of 100%, underscoring its accuracy. Prevalence and predictive values were also determined to reinforce test reliability. Our research highlights the limitations of culture-based screening and supports the suitability of our qPCR-based LDT for GBS detection in a clinical setting.

**IMPORTANCE:**

Rectovaginal colonization by GBS is a major risk factor for early-onset invasive neonatal disease. The most effective approach to reducing the incidence of early-onset disease (EOD) has been described as universal screening, involving assessment of GBS colonization status in late pregnancy and intrapartum antibiotic prophylaxis. Despite its turnaround time and sensitivity limitations, culture remains the gold standard method for GBS screening. However, nucleic acid amplification-based tests, such as qPCR, have been utilized due to their speed and high sensitivity and specificity. This study validated the clinical usefulness of an automated qPCR-LDT for antepartum GBS screening through the Panther Fusion Open Access system (Hologic). Our study addresses the critical need for more robust, sensitive, and rapid strategies for GBS screening in pregnant women that could favorably impact the incidence of EOD.

## INTRODUCTION

Infections caused by *Streptococcus agalactiae*, commonly referred to as group B *Streptococcus* (GBS), are a major cause of neonatal morbidity and mortality, as well as neonatal invasive disease globally (1). When neonatal invasive disease occurs during the first 6 days of life, infected newborns rapidly develop bacteremia, with or without sepsis and/or pneumonia, and, less frequently, meningitis, making it an early-onset disease (EOD). Conversely, when it occurs between days 7 and 89, neonates/infants typically develop bacteremia without focal infections and meningitis, making it a late-onset disease (LOD) (2). The risk factors and transmission routes of LOD are less established, as GBS can be acquired from the mother or from environmental sources (3). However, maternal rectovaginal GBS colonization in late pregnancy is the main risk factor for EOD (4). GBS transiently, intermittently, or persistently colonizes the maternal genital and gastrointestinal tracts and can be transmitted vertically from the colonized mother to the fetus during labor or delivery (5).

Despite the well-documented epidemiology of GBS in developed countries, its impact in developing countries remains unclear. Although regional variations exist (6), approximately 10%–40% of women worldwide are estimated to be colonized by GBS (7). Similarly, estimates of the incidence of EOD differ across countries, with values ranging from 0.02 per 1,000 live births in Southeast Asia to 0–1.5 per 1,000 live births in Africa (1). Probabilistically, in the absence of preventive measures, 40%–60% of neonates exposed during transit through the birth canal are estimated to be colonized; of these patients, 1%–2% will develop EOD (8).

As a maternal vaccine against GBS is currently under development (1, 9), the American College of Obstetricians and Gynecologists (ACOG), and the American Academy of Pediatrics (AAP) recommend prenatal GBS screening and administration of intrapartum antibiotic prophylaxis (IAP) in pregnant women colonized with GBS (2, 10–12), an approach known as universal screening. Although culture using selective enrichment media remains the gold standard method for assessing GBS colonization status (13), its disadvantages, namely sensitivity, labor-intensiveness, and turnaround time, limit its clinical usefulness (14–16). An adequate IAP depends on the timely and accurate assessment of GBS colonization status in pregnant women. False-negative results that are frequent in culture-based methods can delay treatment, thus increasing the risk of severe neonatal and maternal infections, and even death (11, 17). Therefore, it is imperative to develop more sensitive and rapid methods that provide more reliable results to safely prevent EOD and other complications.

Nucleic acid amplification tests (NAATs) have now emerged as the primary research focus for GBS colonization status assessment in pregnant women (18). Polymerase chain reaction (PCR)-based tests, particularly quantitative real-time PCR, have been recommended as they can potentially overcome culture limitations regarding the sensitivity and speed of GBS detection (19). The analytical and clinical performances of quantitative polymerase chain reaction (qPCR) tests of both commercial or laboratory-developed tests (LDTs) must be adequate to rapidly and accurately determine GBS colonization status. This is essential for the successful implementation of IAP, regardless of the differences in terms of its technical requirements (such as degree of automation, turnaround time, infrastructure to minimize contamination risks, and specialized training for operation) (20).

Several NAATs designed for GBS screening in pregnant women are available. The commercial *in vitro* diagnostic (IVD) Panther Fusion GBS assay (Hologic, California, USA) is routinely used in our laboratory but was recently withdrawn temporarily from the market. An LDT validated using the Panther Fusion Open Access system (Hologic) was considered an encouraging strategy to respond to the epidemiological needs of the Dominican population without compromising the speed or reliability of the results and without the obligation to incorporate new technology.

In this study, we described the design and optimization of an LDT TaqMan-qPCR assay in which the *surface immunogenic protein* (*sip*) gene was used as a target for the fully automated detection of *Streptococcus agalactiae* via the Panther Fusion Open Access system (Hologic). We also validated its clinical utility by evaluating the analytic and diagnostic performance of the proposed LDT for GBS screening (LDT-GBS) in late pregnancy using selective-enriched rectovaginal swabs.

## RESULTS

### Oligo design and *in silico* evaluation

When evaluating the homology of the primers and detection probe used in the LDT-GBS assay, all sequences found via Basic Local Alignment Search Tool (BLAST) yielded a coverage and percent identity of >95% and an *E* value less than 10^−2^ only for *S. agalactiae* (GBS) (data not shown). The *in silico* exclusivity of the primers and probe was 100%: sequences in the database of 108 microorganisms biologically and clinically related to *S. agalactiae* yielded a coverage and percent identity less than 95% and an *E* value greater than 10^−2^. Using MFEprimer, v.3.1, the probability of amplifying human DNA sequences (background noise) with the LDT-GBS assay primers was negligible (data not shown).

Of all 9,900 sequences, 18 (0.18%) had only one mismatch in one of the hybridization sequences of the LDT oligonucleotides. No sequences were found with two or more intra- or inter-oligonucleotide mismatches. Only one sequence (0.01%) was found with a mismatch within the last five nucleotides of the hybridization sequence of the 3′-end of the sense primer. Consequently, the calculated coverage (inclusivity) was 99.82% and 99.99%, respectively (Table 1).

**TABLE 1:** *In silico* inclusivity of the primers and detection probe used in the LDT-GBS assay (9,900 sequences)^*a*^

Oligonucleotide	≥1 mismatch in any region of any of the oligonucleotides		≥1 mismatch in the last five nucleotides of the 3′-end/5′-end of any of the oligonucleotides	
	Number of sequences	Relative frequency (%)	Number of sequences	Relative frequency (%)
Forward primer	5	0.05	1	0.01
Reverse primer	9	0.09	0	0.00
TaqMan probe	4	0.04	0	0.00
Total:	18	0.18	1	0.01
Coverage (%)	99.82		99.99	

^*a*^ Note: 9,900 sequences were analyzed from the clinical isolate database PubMLST (www.PubMLST.org). The 3′-end and 5′-end refer to the primers and probe, respectively.

### Amplification efficiency

**Fig 1:**
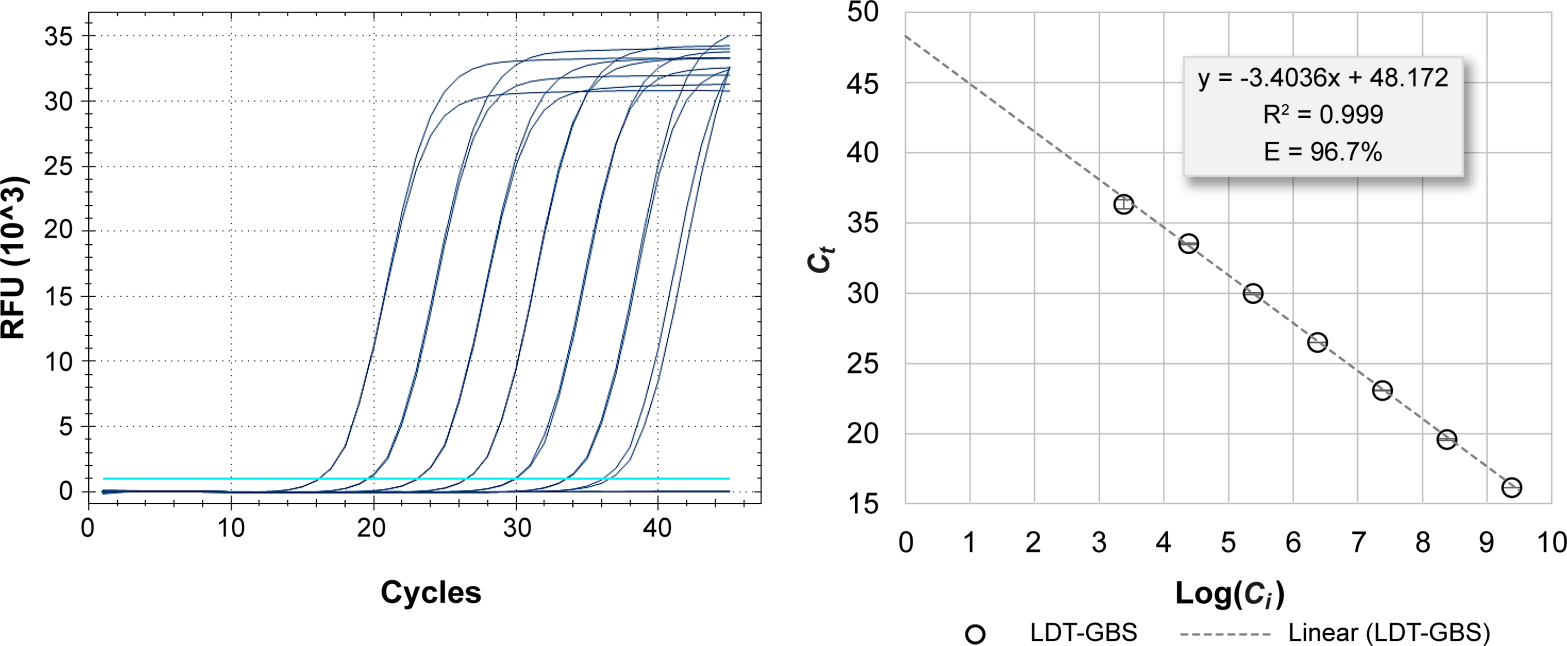
Amplification efficiency of the optimized multiplex LDT-GBS assay. On the left are the fluorescent amplification curves of the panel of seven serial 1:10 dilutions of the synthetic Ultramer duplex control (2.41 × 109–2.41 × 103 copies/mL). On the right is the best linear fit of the *C*_*t*_ plot with respect to the logarithm of the initial nominal concentration (copies/mL) of the dilution panel [log(*C*_*i*_)]. *E*, amplification efficiency; *R*^2^, goodness of fit; RFU, relative fluorescence unit.

The linear fit of the point cloud of cycle threshold (*C*_*t*_) vs the logarithm of the nominal initial concentration of each dilution log(*C*_*i*_) in copies/mL was excellent (*R*^2^ = 0.999). The amplification efficiencies (*E*) of the optimized monoplex and multiplex LDT-GBS assays [in the presence of the template DNA and the Internal Control-X (IC-X) detection primers and probe] were within the 90%–110% range. No significant differences were found between the *C*_*t*_ values obtained for the optimized monoplex and multiplex LDT-GBS assays (|∆*C*_*t*_| ≤ 0.5) (data not shown). The multiplex LDT-GBS assay had an *E* value of 96.7%, considering a fluorescence threshold of 1,000 relative fluorescence unit*s* (RFU) (Fig. 1).

### Analytical performance

**Fig 2:**
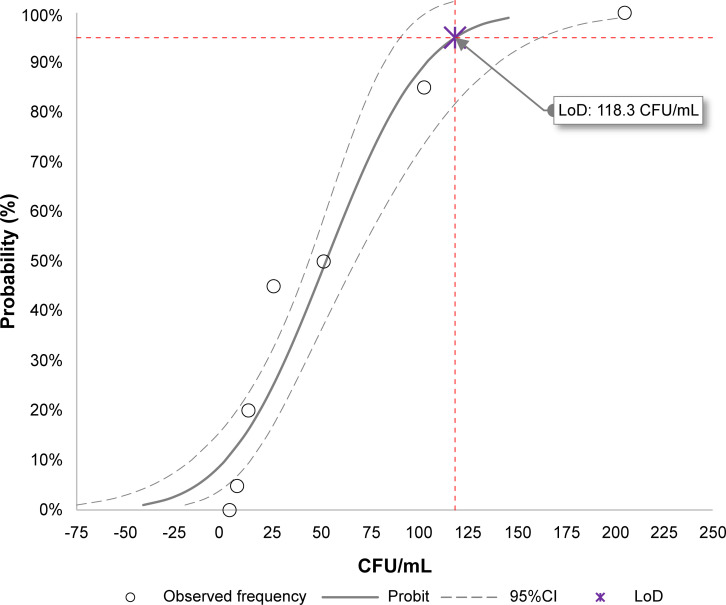
Probit regression: estimation of the limit of detection (LoD) of the LDT-GBS assay in CFU per milliliter. The red dashed lines represent the *C*_95_ value (LoD). The solid and dashed gray lines represent the best fit of the probit regression (theoretical frequency) and its 95% CI, respectively. The circles represent the observed frequency.

Based on probit regression, the analytical sensitivity (LoD) of the LDT-GBS assay was 118 CFU/mL [95% confidence interval (CI): 96–162 CFU/mL] (Table 2; Fig. 2).

**TABLE 2:** Hit rate per dilution of the panel for the analytical sensitivity evaluation of the LDT-GBS assay

Dilution	Analyte concentration (CFU/mL)	Replicates	Observed frequency	Hit rate (%)
1	205.13	20	20	100
2	102.56	20	17	85
3	51.28	20	10	50
4	25.64	20	9	45
5	12.82	20	4	20
6	6.41	20	1	5
7	3.21	18	0	0

**Fig 3:**
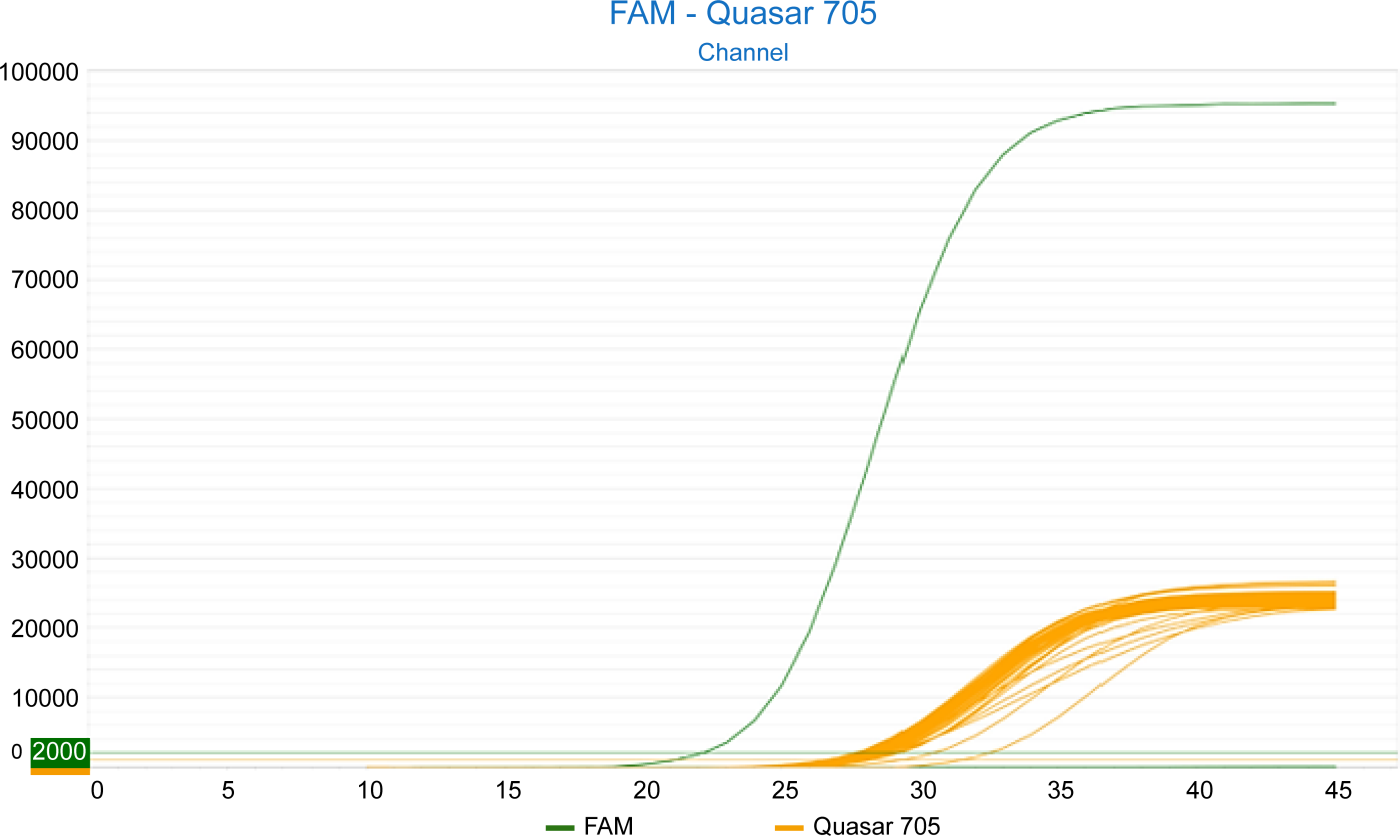
Fluorescence amplification curves of the analytical specificity evaluation (cross-reactivity). The fluorescence amplification curve in green corresponds to the positive control (*S. agalactiae* serotype Ia, ATCC 12386) (FAM channel). The fluorescence amplification curves of IC-X (Quasar 705 channel) are shown in orange.

The analytical specificity (cross-reactivity) of the LDT-GBS assay was evaluated for 67 pathogens. No sigmoid amplification curve was observed for any of the replicates of the samples containing the pathogen, except for the positive control consisting of *S. agalactiae* serotype Ia [American Type Culture Collection (ATCC) 12386] (Fig. 3). Therefore, no cross-reactivity was observed for any of the 67 pathogens clinically and biologically related to *S. agalactiae* that were used in the evaluation.

### Agreement between qPCR-based assays

No exclusions occurred, as no invalid results were obtained by any of the assays included in the evaluation. The LDT-GBS assay yielded only one discordant positive result (0.3%) compared with the Panther Fusion GBS comparative assay and one discordant negative result (0.3%) compared with the VIASURE *Streptococcus* B Real-Time comparative assay (VIASURE GBS) (Table 3). Both samples were negative according to culture. Therefore, the overall percent agreement (OPA) was 99.7% (95% CI: 98.1%–99.9%), with *k* = 0.992 (95% CI: 0.975–1.000) in both comparison pairs (Table 3). These results agree with those of the comparative commercial qPCR-based assays, Panther Fusion GBS and VIASURE GBS.

**TABLE 3:** Agreement of the LDT-GBS assay with the comparative qPCR-based assays (*n* = 300)^*a*^

Comparative qPCR-based assays		LDT-GBS			Agreement		95% CI
		–	+	Total			
Panther Fusion GBS	–	218 (72.7%)	1 (0.3%)	219 (73.0%)	NPA	0.995	0.975–0.999
					PPA	1.000	0.955–1.000
	+	0 (0.0%)	81 (27.0%)	81 (27.0%)	OPA	0.997	0.981–0.999
					*k*	0.992	0.975–1.000
Total		218 (72.7%)	82 (27.3%)	300 (100%)			
VIASURE *Streptococcus* B real time	–	217 (72.3%)	0 (0.0%)	217 (72.3%)	NPA	1.000	0.983–1.000
					PPA	0.988	0.935–0.998
	+	1 (0.3%)	82 (27.3%)	83 (27.7%)	OPA	0.997	0.981–0.999
					*k*	0.992	0.975–1.000
Total		218 (72.7%)	82 (27.3%)	300 (100%)			

^*a*^ CI, confidence interval; *k*, Cohen’s kappa coefficient; NPA, negative percent agreement; OPA, overall percent agreement; PPA, positive percent agreement.

### Diagnostic performance

No exclusions occurred, as no invalid results were obtained by any of the assays included in the evaluation. The positive rates (PRs) were 14.7% (95% CI: 10.7%–19.2%), 27.7% (95% CI: 22.7%–33.1%), 27.0% (95% CI: 22.1%–32.4%), and 27.3% (95% CI: 22.4%–32.8%) for the comparative assays (culture, VIASURE GBS, and Panther Fusion GBS) and the assay under evaluation (LDT-GBS), respectively. According to the nonparametric McNemar-Mosteller statistical test, the differences in PR between the culture and qPCR tests, including the LDT-GBS assay, were statistically significant (*P* < 0.0001). However, PR differences between the LDT-GBS assay and the comparative qPCR assays were not statistically significant (*P* = 0.3137). Regarding consensus, 38 (12.7%) and 1 (0.3%) false negatives were found when using the culture and the Panther Fusion GBS assays, respectively, and 1 (0.3%) false-positive result was found when using the VIASURE GBS assay. All LDT-GBS assay results were concordant with the consensus (Table 4).

**TABLE 4:** Diagnostic performance: Diagnostic sensitivity and specificity of the LDT-GBS compared with the consensus (*n* = 300)^*a*^

Assay	TP	FP	FN	TN	*n*	PR (%) (95% CI)	Sensitivity (%) (95% CI)	Specificity (%) (95% CI)
Culture/MALDI-TOF	44	0	38	218	300	14.7 (10.7%–19.2%)	53.7 (42.9%–64.0%)	100.0 (98.3%–100.0%)
VIASURE GBS (CerTest)	82	1	0	217	300	27.7 (22.7%–33.1%)	100.0 (95.5%–100.0%)	99.5 (97.4%–99.9%)
Panther Fusion GBS (Hologic)	81	0	1	218	300	27.0 (22.1%–32.4%)	98.8 (93.4%–99.8%)	100.0 (98.3%–100.0%)
LDT-GBS	82	0	0	218	300	27.3 (22.4%–32.8%)	100.0 (95.5%–100.0%)	100.0 (98.3%–100.0%)

^*a*^ CI, confidence interval; FN, false negative; FP, false positive; MALDI-TOF, matrix-assisted laser desorption/ionization-time of flight; *n*, total number of samples; PR, positive rate; TN, true negative; TP, true positive.

The diagnostic sensitivity of the LDT-GBS assay was 100% (95% CI: 95.5%–100%). Results were not significantly different from the diagnostic sensitivities of the comparative qPCR tests VIASURE GBS and Panther Fusion GBS: 100% (95% CI: 95.5%–100%) and 98.8% (95% CI: 93.4%–99.8%) (*P* = 0.3173), respectively. However, the diagnostic sensitivity of culture, 53.7% (95% CI: 42.9%–64.0%), was significantly lower than that of qPCR assays, including the LDT-GBS assay (*P* < 0.0001). The diagnostic specificity of the LDT-GBS assay was 100% (95% CI: 98.3%–100%). Statistically, this was not significantly different from the diagnostic specificity of the comparative tests: culture, 100% (95% CI: 98.3%–100%); VIASURE GBS , 99.5% (95% CI: 97.4%–99.9%) (*P* = 0.3173); and Panther Fusion GBS, 100% (95% CI: 98.3%–100%) (Table 4).

Considering the consensus and population sample size (*n* = 285), we found that the prevalence of GBS colonization in pregnant women was 25.3% (95% CI: 20.3%–30.7%). Using culture exclusively, we found that the prevalence (measured as PR) was 12.6% (95% CI: 9.0%–17.1%) (Table 5). According to the nonparametric McNemar-Mosteller statistical test, the difference between the prevalence based exclusively on the culture and the prevalence based on the consensus was statistically significant (*P* < 0.0001). Moreover, Pearson’s chi-square test indicated that the difference between the prevalence based on the consensus and the expected prevalence (44%) (21) was also statistically significant (*P* < 0.0001).

**TABLE 5:** Diagnostic performance: positive and negative predictive values of the LDT-GBS compared with the consensus (clinical samples only: *n* = 285)^*a*^

Assay	TP	FP	FN	TN	*n*	PR (%) (95% CI)	PPV (%) (95% CI)	NPV (%) (95% CI)
Culture/MALDI-TOF	36	0	36	213	285	12.6 (9.0%–17.1%)	50.0 (42.5%–57.5%)	100 (98.3%–100.0%)
VIASURE GBS (CerTest)	72	0	0	213	285	25.3 (20.3%–30.7%)	100.0 (95.0%–100.0%)	100 (98.3%–100%)
Panther Fusion GBS (Hologic)	71	0	1	213	285	24.9 (20.0%–30.4%)	98.6 (91.0%–99.8%)	100 (98.3%–100%)
LDT-GBS	72	0	0	213	285	25.3 (20.3%–30.7%)	100.0 (95.0%–100.0%)	100 (98.3%–100%)

^*a*^ CI, confidence interval; FN, false negative; FP, false positive; *n*, total number of samples; NPV, negative predictive value; PR, positive rate; PPV, positive predictive value; TN, true negative; TP, true positive.

## DISCUSSION

Several genetic markers have been used to detect *S. agalactiae* (20, 22–24). The risk of false negatives has recently been described in some regions of the world, following the identification of four types of chromosomal deletions in some GBS isolates affecting the *cfb* gene (25, 26), which is the most commonly used target for GBS screening in pregnant women (6, 27). The *sip* gene, which encodes the surface immunogenic protein, has garnered considerable attention in recent years as a potential target for use as a GBS detection marker (28–32), presumably because of its low variability among known *S. agalactiae* serotypes (33). The development of a fully automated *sip*-targeted qPCR-based test employing the Panther Fusion Open Access system (Hologic) was a strategy for optimizing performance, resources, and technology, given the temporary withdrawal of the commercial Panther Fusion GBS (Hologic) assay from the market.

The design of the oligonucleotides for the LDT described in this study focused on the *S. agalactiae sip* gene. The percentage of design coverage was calculated as an estimator of the *in silico* inclusivity of the LDT-GBS assay and was greater than 99%. Given the lack of more than one intra- and/or inter-oligonucleotide hybridization site mutation, those results indicate a low probability of false negatives due to limited inclusivity. This is further reinforced by the fact that most identified mutations were found in sequences that hybridized before the last five nucleotides of the 3′-end of the primers. Similarly, the evaluation of the *in silico* exclusivity of the LDT-GBS assay led to the identification of four elements indicative of a very low risk of false positives due to cross-reactivity: high homology with sequences of the GBS *sip* gene, low homology with sequences of microorganisms biologically and clinically related to GBS, low homology with human genome sequences, and low or no probability of amplification of human DNA (background noise). The *in vitro* analytical specificity evaluation results confirm these predictions: The LDT-GBS assay did not exhibit cross-reactivity against any of the 67 pathogens tested clinically and biologically related to GBS, consistent with an excellent analytical specificity of the LDT.

An essential disadvantage of LDT optimization on the Panther Fusion Open Access system is the low yield of primer and probe reconstitution solutions (PPRs). Various combinations of different amplification mixture component concentrations (PPRs), conveniently distributed on the instrument workbench, are required. Each PPR typically requires 33%–70% more reactions than necessary, depending on the number of PPR tests, to accommodate the imprecision of robotic liquid handling in the automated system. To improve the yield, we performed the LDT-GBS assay optimization process using the CFX96-IVD analyzer (Bio-Rad, California, USA), a third-party instrument equivalent in terms of the number and compatibility of the detection channels. The optimal amplification efficiency confirmed the effectiveness of this approach. Although this is a novel strategy for LDT optimization for the Panther Fusion Open Access system, it has been recommended for open channels of other platforms, such as the cobas 5800/6800/8800 system (34, 35).

The analytical sensitivity or LoD of the LDT-GBS assay was comparable to that reported by the manufacturers of the commercial Panther Fusion GBS (Hologic) (36) and BD MAX GBS (BD, Maryland, USA) (37) assays, for GBS serotype Ia. Similarly, when considering a study by Thwe et al. (22), the LoD of the LDT-GBS assay and its 95% CI were within the LoD intervals reported for the Panther Fusion GBS (Hologic) and Xpert GBS LB XC (Cepheid, California, USA) assays: 84–301 CFU/mL and 40–682 CFU/mL, respectively. However, compared with those of the commercial Xpert GBS LB (Cepheid) and ARIES GBS (Luminex—DiaSorin, Texas, USA) assays, the LoD of the LDT-GBS assay was 10 and 100 times lower (more sensitive), respectively (38).

The comparison of the LDT-GBS assay with its commercial counterparts, Panther Fusion GBS (Hologic) and VIASURE *Streptococcus* B Real-Time (CerTest Biotec, Zaragoza, Spain) showed few disagreements. The positive and negative disagreements found in the LDT-GBS assay are attributed to a false negative and a false positive, respectively, in the Panther Fusion GBS and the VIASURE *Streptococcus* B Real-Time comparative assays. This was confirmed by reprocessing all assays included in the evaluation and comparing the results with the consensus. In the first case, the false negative was attributed to the analysis and interpretation rules of the commercial Panther Fusion GBS assay. In this respect, a fluorescent amplification curve exceeding the fluorescence threshold could be visualized, but with a *C*_*t*_ value greater than the cutoff *C*_*t*_ defined by the test manufacturer. In the second case, although the false positive of the VIASURE *Streptococcus* B Real-Time assay could be attributed to a lower limit of detection, the discordant sample originated from a round (D8-C 2021) of the College of American Pathologists (CAP)’s external quality assessment program, where 97.9% of the participating laboratories reported a negative result, following the expected result given by the provider. Despite these findings, the results of the LDT-GBS assay agreed favorably with the results of the comparative qPCR assays, which was supported by a high OPA and Cohen’s kappa coefficient (*k*) in both cases. These results are consistent with those of previous studies, in which the OPA between NAATs ranged from 92% to 98.4% (24, 38–40). The high degree of agreement between the qPCR assays corroborates the commutability of the LDT-GBS assay with its commercial counterparts evaluated in this study.

The diagnostic performance of the LDT-GBS assay was evaluated against a consensus based on the results of the reference method (culture), the two commercial qPCR-based assays, and the LDT under evaluation. In this scenario, the diagnostic sensitivity of the LDT-GBS assay was excellent. There were no significant differences from those estimated for the comparative qPCR tests VIASURE *Streptococcus* B Real-Time and Panther Fusion GBS (*P* = 0.3173). However, the sensitivity of the culture was significantly lower (*P* < 0.0001) than that of all the qPCR tests evaluated, including the LDT-GBS assay; the culture was 46.3% less sensitive than the LDT-GBS and comparative qPCR assays. Logically, the differences between the PRs of the different assays exhibited the same behavior. In contrast, the diagnostic specificity of the LDT-GBS was 100%, which was significantly different neither from those of the commercial qPCR assays, VIASURE *Streptococcus* B Real-Time (*P* = 0.3173) and Panther Fusion GBS, nor from the culture, as expected. These results are consistent with those of previous studies. For example, Shin and Pride (38), Thwe et al. (22), and de-Paris et al. (41) reported an increase of 25.0%–40.9% in the sensitivity of GBS detection using qPCR when compared with the culture. According to Miller et al. (24), the sensitivity of qPCR-based tests ranged from 90.9% to 100%, which was 37.3%–46.4% greater than that of culture (53.6%). In our study, although the improvement in the sensitivity of the LDT-GBS assay (and that of the comparative qPCR assays) is consistent with the latest available report (24), the sensitivity of the culture was also within the commonly reported range in the literature: 53%–70% (13). The diagnostic sensitivity and specificity of our LDT-GBS assay (and the comparative qPCR assays) when compared with the consensus were within the weighted ranges 93.3%–100.0% and 95.8%–100.0%, respectively (Table S1, Fig. S1, and Fig. S2) (17, 22, 24, 32, 38, 42–44). These findings place the diagnostic performance of most qPCR-based tests, including the LDT-GBS, above the predefined cutoff values (90% for sensitivity and specificity) (13, 19).

According to the ACOG and AAP guidelines, all pregnant women should be screened for GBS between 36 0/7 and 37 6/7 weeks of gestation (11, 12). The maternal GBS colonization status determines the decision that should be made when administering IAP. This strategy reduces the number of inappropriate antimicrobial treatments aimed at preventing the onset of neonatal invasive disease caused by GBS, reduces the costs of treating newborns potentially infected by GBS (45), and contributes to reducing the development of chronic diseases (such as obesity, allergies, infections, and inflammatory or brain disorders) related to disrupting the establishment of the intestinal microbiota in the first days after birth (44). One study reported that antepartum screening can reduce the use of antibiotics by approximately 40% without increasing the risk of infection for the mother or neonate (46). For these reasons, when GBS screening is solely dependent on culture, the high prevalence and underestimation of GBS colonization status highlight the need for faster, more robust, and more sensitive strategies.

NAATs can be exclusively used only when culture is unavailable (6), but culture combined with NAATs is the setting that most favorably impacts the incidence of neonatal invasive disease (45). Despite its suboptimal sensitivity, culture can be used to evaluate antimicrobial susceptibility in pregnant women at risk of anaphylaxis due to penicillin (4, 5, 44). Conversely, given their high sensitivity and speed, NAATs can reduce the risk of false negatives while improving the timeliness of the results without significantly compromising specificity (19, 24). Although a combination of methods is possible, the need to adopt NAATs as the new gold standard is becoming increasingly apparent. The first steps in that direction are corroborated by studies that highlight the potential usefulness of the simultaneous detection of GBS with certain markers of genotypic antimicrobial resistance (47–49). Additionally, situations in which numerous patients require GBS screening, the ease of scaling and automation capability of NAATs becomes desirable. The development of diagnostic tests in the laboratory and their validation in *in vitro* diagnostic platforms improve the response capacity of the clinical laboratory to local epidemiological needs. The use of automated instruments, such as Panther Fusion, maximizes confidence in the results and processing efficiency while minimizing laboratory turnaround time.

Overall, 10%–40% of women worldwide are colonized by GBS, ranging from very low values in Asia (10%–14%) to very high values in the Caribbean (35%–40%) (6, 7, 50). In 2006, Fernández et al. (21) reported a prevalence of GBS colonization as high as 44% in the Dominican Republic. This finding suggested that the prevalence of GBS colonization and, therefore, the incidence of neonatal invasive disease in the Dominican Republic could be the highest in the region.

To the best of our knowledge, this is the first study in the Dominican Republic to develop and validate a qPCR assay using an *in vitro* diagnostic platform. This study is also the first to evaluate the prevalence of GBS colonization in the Dominican Republic using the consensus between three NAATs and culture/matrix-assisted laser desorption/ionization-time of flight (MALDI-TOF). Therefore, the prevalence estimated with such analytical rigor using multiple methods and assays should be more accurate. The difference between the prevalence estimated exclusively based on the culture and that calculated based on the consensus was statistically significant (*P* < 0.0001), with the first (12.6%) being 50.2% lower than the second (25.3%). Conversely, the difference between the prevalence calculated based on the consensus and the expected prevalence (44%), as indicated by Fernández et al. (21), was significant (*P* < 0.0001), even though the reference study used culture as the exclusive method for the evaluation of GBS colonization. The first difference (culture-based vs consensus-based prevalence) directly results from differences in sensitivity between the culture and qPCR-based tests, as previously discussed. The second difference (consensus-based vs expected prevalence) could be attributed to differences in, but not limited to, (i) the period in which the studies were performed; (ii) the size of the population sample, its representativeness, and the homogeneity of the distribution between different social strata, risk groups, and national or regional geography; (iii) the inclusion and exclusion criteria, if any; (iv) the level of knowledge and rigor in complying with guidelines that govern good practices and quality assurance in the microbiology laboratory; (v) the regulations and level of compliance with over-the-counter sales of antibiotics; and (vi) the analytical method, the method, and anatomical location of sample collection (anal, vaginal, or both) (32, 51, 52). Although the calculated prevalence was lower than that reported for the Caribbean (35%–40%) (50), it remains higher than that reported in regions with more economic development, such as Europe, Australia, and North America, which range from 15% to 21% (50).

Our study has three main limitations. First, the study was performed in a single health center (monocentric study), and the sample size was likely not representative of the national territory, social strata, or risk groups: most samples were obtained from Santo Domingo city and Distrito Nacional (National District). Second, as the samples used for the LDT-GBS assay validation were residual clinical samples, only analytical acceptance and rejection criteria were applied. Therefore, data on conditioning variables, such as recent antimicrobial treatment during sample collection or related to other risk factors, such as preterm birth, multiple pregnancies, rupture of membranes lasting for >18 h, or type of delivery, could not be collected. Finally, due to availability limitations, the LoD and inclusivity of the LDT-GBS assay were evaluated only for *S. agalactiae* serotype Ia.

### Conclusions

The excellent analytical and clinical performance of the LDT-GBS assay makes it suitable for the fully automated antepartum assessment of GBS colonization status in pregnant women at ≥35 (36 0/7) weeks of gestation. Given its excellent agreement with commercial *in vitro* diagnostic tests, Panther Fusion GBS and VIASURE *Streptococcus* B Real-Time, the LDT-GBS assay is a promising (commutable) alternative. This study demonstrated that GBS detection by culture poses a very high risk to the safety of newborns. Finally, employing a Panther Fusion compatible real-time PCR analyzer, such as CFX96-IVD, during the LDT optimization stage likely increases the PPR yield while being time efficient.

## MATERIALS AND METHODS

### Clinical samples, ethical aspects, and eligibility

The methodological approach described in this article was performed by the Research and Development Group of the Molecular Biology Department of the Referencia Laboratorio Clínico (Headquarters) located in Santo Domingo Oeste, Dominican Republic, from April 2022 to June 2023. The minimum sample size was estimated using the ‘Estimate proportion (random sampling and perfect diagnostic)’ option of the web application WinEpi, v.2.0 (http://www.winepi.net/winepi2/f102.php, accessed on 5 April 2022) (53), considering a confidence level of 95%, an acceptable error (desired precision) of 10%, an unknown population size, and an expected prevalence of 44% (21). The minimum sample size estimated for a binomial distribution was 93 clinical samples or individuals.

The biological samples used in the evaluation were obtained from two sources: (i) remnants of enrichment broths from pregnant women with ≥36 0/7 weeks of gestation with medical indications for GBS screening by culture (*n* = 285), and (ii) samples with results assigned by a consensus from three rounds (D8-B 2021, D8-C 2021, and D8-A 2022) of an external quality assessment of the CAP (*n* = 15), for a total of 300 biological samples. The clinical samples were collected from 14 provinces and the National District of the Dominican Republic.

Primary clinical samples [rectovaginal swabs preserved in liquid Amies medium without charcoal, eSwab (Copan, Brescia, Italy)] were used to evaluate the agreement and diagnostic performance of the laboratory-developed test for GBS screening (LDT-GBS assay). The samples were subsequently transported to the clinical laboratory at 2°C–8°C within 12 h after collection. All clinical samples and samples with consensus-assigned results were placed in 5 mL of Lim broth (30 g/L of Todd Hewitt broth, 10 g/L of yeast extract, 15 mg/L of nalidixic acid, and 10 mg/L of colistin sulfate) (Hardy Diagnostics, Santa María, CA, USA) for selective enrichment and then incubated at 37°C for 18–24 h under aerobic conditions. Enriched samples were immediately tested using the LDT-GBS assay (candidate assay) and comparative assays: culture (reference method) and two commercial qPCR-based assays: Panther Fusion GBS (Hologic) and VIASURE *Streptococcus* B Real-Time (CerTest Biotec).

Clinical samples were collected in compliance with the confidentiality and informed consent policies of Referencia Laboratorio Clínico. All patients agreed with the potential use of the remnants of the clinical samples (residual clinical samples) in research and development and the possible publication of the corresponding results. No rejection criteria were applied at the preanalytical stage. The median age of the pregnant women who participated in the study was 31 years old (17–46 years old).

### Oligo design

**Fig 4:**
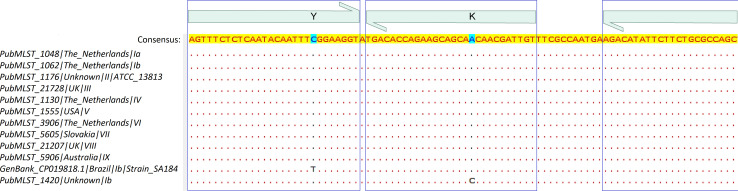
Target sequence of the *surface immunogenic protein* gene (amplicon: 90 bp) of the primers and the detection TaqMan probe of the laboratory-developed test for GBS screening (LDT-GBS). The primers (right and left ends) and the probe (center) are shown in green, while the consensus sequence is in yellow.

Two thousand and eighty-one *S. agalactiae sip* gene sequences were retrieved from the Bacterial and Viral Bioinformatics Resource Center (https://www.bv-brc.org) database. The sequences were aligned using the MAFFT algorithm of Unipro UGENE software, v.48.0 (Unipro, Novosibirsk, Russia), and a conserved region that did not exclude the well-known serotypes Ia–IX was located (Fig. 4).

Two primers and a detection TaqMan probe (Table 6) were designed by combining Oligo, v.7.60 (Molecular Biology Insights, Inc., Colorado, USA), Benchling (Benchling, California, USA), and the OligoAnalyzer tool [Integrated DNA Technologies (IDT), Iowa, USA]. As a reference, the consensus sequence resulting from the alignment of the representative *sip* gene sequences corresponding to serotypes Ia–IX of *S. agalactiae* was used (Fig. 4; Table S2). The 5′-end of the detection TaqMan probe was labeled with the fluorophore 6-carboxyfluorescein, and the 3′-end was phosphorylated and labeled with the quencher Iowa Black FQ. Nucleotide 9 (5′→3′) was labeled with the internal quencher ZEN (Table 6).

**TABLE 6:** Primers and the detection probe of the LDT-GBS assay

Oligonucleotides		Tm (°C)^*a*^	% GC^*b*^	Length (bp)
Type	Sequence (5′→3′)			
Forward primer	AGT TTC TCT CAA TAC AAT TTY GGA AGG T	64.4–65.5	33.9–35.7	28
Reverse primer	GCT GGC GCA GAA GAA TAT GTC T	64.7	50.0	22
TaqMan probe	6-FAM/CA ATC GTT G/ZEN/K TGC TGC TTC TGG TGT CA/IABkFQ^*d*^	69.5–70.7	48.2–50.0	27
Ultramer duplex control^*c*^	*GTT TCT GTT GCA GAC CAA AA*A GTT TCT CTC AAT ACA ATT TCG GAA GGT A**TG ACA CCA GAA GCA GCA ACA ACG ATT G**TT TCG CCA ATG AAG ACA TAT TCT TCT GCG CCA GC*T TTG AAA TCA AAA GAA GTA TT*			

^*a*^ The melting temperature (Tm) of the oligos was estimated using OligoAnalyzer (IDT, Iowa, USA) according to the algorithm published by SantaLucia (54), which considers the following assumptions: oligo concentration, 200 nM; divalent ion concentration, 3 mM; monovalent ion concentration, 50 mM; deoxynucleotide triphosphate concentration, 0.8 mM.

^*b*^ Guanine-cytosine pair content (%).

^*c*^ Nucleotide sequence of the Ultramer duplex synthetic control containing the target sequence (amplicon) of the LDT-GBS assay. The target sequences of the sense and antisense primers are underlined. The target sequence of the detection TaqMan probe is shown in bold. The sequences flanking the target sequence are shown in italics*.*

^*d*^ 6-FAM, 6-carboxyfluorescein; IABkFQ, Iowa Black FQ.

An oligonucleotide containing the 131-bp sequence of the *S. agalactiae* serotype Ia (GenBank accession number DQ914240.1) of the *sip* gene was used as an amplification control for all optimization and validation assays. This sequence comprises the target sequence of the LDT-GBS assay (amplicon: 90 bp) plus two additional flanking sequences: 20 bp upstream of the 5′-end of the sense primer and 21 bp downstream of the 5′-end of the antisense primer (Table 6).

All oligonucleotides (primers, TaqMan probe, and Ultramer duplex control) were synthesized at IDT and purified using high-performance liquid chromatography. The optimal primer and probe purity were defined as ≥85%. An amount of 4 nmol was purified by standard desalting of the synthetic control via the Ultramer duplex DNA Oligos service. All oligonucleotides were ordered lyophilized.

### *In silico* specificity evaluation

The *in silico* specificity of the primers and TaqMan probe was analyzed by evaluating the homology, the inclusivity of known *S. agalactiae* serotypes, and the exclusivity of other related microorganisms and human DNA background noise.

Homology analysis was performed using the BLAST of the GenBank database (https://blast.ncbi.nlm.nih.gov). High-homology sequences were defined as those with a percentage of identity and coverage of ≥95% and an *E* value less than or equal to 10^−2^. Exclusivity was evaluated using a similar approach, conducting a homology search among the existing sequences of at least 108 microorganisms clinically and biologically related to *S. agalactiae* (Table S3). A low probability of false positives was attributed to any sequence meeting at least two of the following criteria: (i) identity percentage of less than 95%, (ii) coverage percentage of less than 95%, and (iii) *E* value greater than 10^−2^. Cross-reactivity against human DNA (background noise) was evaluated using the MFEprimer web tool, v.3.1 (https://mfeprimer3.igenetech.com/). For this purpose, the background noise database “UCSC-Homo sapiens-hg38–Genome” was selected, with all the other parameters set to the default settings, except for the MgCl_2_ concentration, which was set to 3 mM.

Inclusivity was evaluated using a coverage analysis. For this purpose, 9,900 *sip* gene sequences from clinical isolates of all known *S. agalactiae* serotypes (serotypes Ia–IX) stored in the Public Databases for Molecular Typing and Microbial Genome Diversity (www.PubMLST.org) from 1953 to 2022 were aligned. The alignment was performed using the MAFFT algorithm of Unipro UGENE, v.4.8.0 (Unipro). Coverage was calculated as the relative number of published sequences containing no mutations in the hybridization sites of the oligonucleotides with respect to the total number of sequences used. For this purpose, the following parameters were calculated: (i) the percentage of sequences with mutations (mismatch) in any hybridization sites region of any of the oligonucleotides and (ii) the percentage of sequences with mutations only in the last five nucleotides of the 3′-end of any of the primers or the 5′-end of the probe. Both measurements were sorted by the number of mutations in the same sequence (inter- and intra-oligonucleotide).

### Panther Fusion Open Access RNA/DNA Enzyme Cartridge: Off-Label Protocol

Optimization assays and amplification efficiency evaluation were performed with the Panther Fusion Open Access RNA/DNA Enzyme Cartridge (Hologic) in 96-well amplification plates using the CFX96-IVD analyzer (Bio-Rad) (off-label use). Although the general enzyme manufacturer instructions that guarantee optimal performance were followed, some modifications were made for its use in the CFX96-IVD analyzer. Briefly, the cartridge wells were punched, and the lyophilized enzyme was reconstituted with 20 µL of the primer and probe reconstitution solution (PPR), incubated at room temperature (18°C–25°C) for 1–5 min, and vortexed using short and repeated pulses avoiding turbulence. Amplification was performed in a reaction volume of 25 µL [20 µL of the amplification mixture (master mix) + 5 µL of template DNA].

### LDT assay optimization

The concentrations of the oligonucleotides (primers and probe) and salts (KCl and MgCl_2_) were optimized, as was the annealing temperature (Ta) of the amplification and detection protocol of the LDT-GBS assay. The other amplification protocol variables (denaturation temperature and time, annealing time, and number of amplification cycles) were set to the default settings of the standard DNA amplification protocol of MyAccess software (Hologic); this ensured that the permitted amplification time (55 min) was not affected.

The concentrations of the salts (KCl and MgCl_2_) were optimized simultaneously. For this purpose, both primers (1:1) and the detection probe were used at baseline concentrations of 0.6 and 0.3 µM, respectively. Then, 25 PPRs were prepared corresponding to all possible combinations of the following concentrations: 0-, 2-, 3-, 4-, and 5-mM MgCl_2_ and 0-, 25-, 50-, 75-, and 100-mM KCl. The same procedure was used for primers and probe concentration optimization at the optimal salt concentrations. For this purpose, 12 PPRs were prepared for all possible combinations of the following concentrations: 0.4-, 0.6-, 0.8-, and 1.0-µM primer mixture (1:1) and 0.2-, 0.4-, and 0.6-µM TaqMan probe. All PPR solutions were prepared in 8-mM Tris-HCl buffer. Ta was optimized using a temperature gradient amplification protocol in the CFX96-IVD analyzer. The optimal concentrations of salts and oligonucleotides were used, and the analyzer manufacturer’s recommendations were followed. The Ta gradient was set in the range of 10°C, from 56°C to 66°C (56.0°C, 56.7°C, 58.0°C, 60.0°C, °C62.4°C, 64.3°C, 65.5°C, and 66°C). All optimization assays were performed with the Ultramer duplex synthetic control at a nominal concentration of 2.41 × 10^5^ copies/mL (*C*_*t*_ ≈ 30). The optimal conditions for the LDT-GBS assay are summarized in Tables 7 and 8.

**TABLE 7:** Optimized PPR for the LDT-GBS assay

Component	Final concentration
KCl	100.0 mM
MgCl_2_	3.0 mM
Tris-HCl	8.0 mM
GBS forward primer	0.60 µM
GBS reverse primer	0.60 µM
GBS probe	0.40 µM
DNA IC primer mix^*a*^	0.30 µM
DNA IC probe^*b*^	0.20 µM

^*a*^ Primer mix for the detection of the Panther Fusion Internal Control-X (IC-X).

^*b*^ TaqMan probe for the detection of the Panther Fusion (IC-X).

**TABLE 8:** Optimized amplification/detection protocol for the LDT-GBS assay

Step	Temperature (°C)	Time	Cycles
Activation	95	2 min	1
Amplification/detection	95	8 s	45
	60^*a*^	25 s	

^*a*^ Fluorescence reading.

### Amplification efficiency and multiplex compatibility

A panel of eight 1:10 serial dilutions of the Ultramer duplex synthetic control was prepared in Tris-ethylenediaminetetraacetic acid (EDTA), pH 8.0 (10-mM Tris and 0.1-mM EDTA) (IDT). The dilution at the highest concentration was prepared at a nominal concentration of 2.41 × 10^9^ copies/mL. Each level of the amplification efficiency evaluation panel was amplified in duplicate using the optimized LDT-GBS assay (Tables 7 and 8). Amplification was performed using the Panther Fusion Open Access RNA/DNA Enzyme Cartridge (Hologic) on the CFX96-IVD analyzer (Bio-Rad); refer to Panther Fusion Open Access RNA/DNA Enzyme Cartridge: Off-Label Protocol.

In a plot of the logarithm of the relative fluorescence unit vs amplification cycles, the fluorescence threshold was set at the midpoint of the linear phase of the amplification curves (1,000 RFU). The *C*_*t*_ values were plotted against the log(*C*_*i*_). The point cloud was fitted to a curve with the general equation *y = mx +* n, where *y* is the *C*_*t*_ value; *x* is the log(*C*_*i*_) value; *m* is the slope; and *n* is the intersection with the *y*-axis (linear regression). The slope and goodness of fit (*R*^2^) were calculated. The amplification efficiency (*E*) was calculated using the following equation:


(i) $$E=\left[10^{\left(-1/m\right)}-1\right]\times 100\%$$

The interference of concurrent amplification of the Panther Fusion IC-X and the GBS *sip* gene target sequence—multiplex compatibility—was evaluated. Following the same amplification efficiency evaluation approach, the *C*_*t*_ values and amplification efficiencies of the LDT-GBS assay were calculated in the absence (monoplex) and presence (multiplex) of DNA template and IC-X analyte-specific reagents (DNA IC Primer Mix and DNA IC Probe). Multiplex compatibility was assumed when the |△*C*_*t*_| was less than or equal to 0.5 between the monoplex and multiplex LDT-GBS assays at all standard curve dilutions. A range of 90%–110% was regarded as an acceptable *E*. The fluorescent signal of the IC-X detection probe was read on the Quasar 705 channel.

### Analytical sensitivity (limit of detection) and cutoff

The LoD was calculated and expressed in colony-forming units per milliliter using serial dilutions of *S. agalactiae* serotype Ia (ATCC 12386). Briefly, the bacterial suspension turbidity was adjusted to the 0.5-McFarland standard, and six 1:10 serial dilutions were prepared with isotonic saline solution (BioMérieux SA, Marcy-l'Étoile, France). Then, 100 µL of each dilution was plated in triplicate on blood agar, and dilutions with 30–300 colonies in all replicates were selected. The colonies were counted and averaged between replicates, and the concentration in CFU per milliliter was calculated. Next, the analytical sensitivity evaluation panel was prepared using additional 1:2 serial dilutions. As a diluent, Lim broth (Hardy Diagnostics) was diluted in Aptima Specimen Transfer medium (Hologic) according to the manufacturer’s recommended ratio (1:3.9) (55). At least 18 replicates of at least seven dilutions (3.21–205.13 CFU/mL) of the panel were analyzed using the LDT-GBS assay. The LoD and 95% CI were calculated using probit regression in SPSS software, v.25 (IBM Corp., New York, USA). The LoD was defined as the concentration corresponding to a probability of occurrence or success of 95% (*C*_95_).

The maximum *C_t_* value of the LoD experiment plus 1 [cutoff = max(*C*_*t*_) + 1] was assumed as the cutoff point. Thus, the cutoff used in subsequent experiments was set at *C*_*t*_ = 39.0 (data not shown) (Fig. S3).

### Analytical specificity (cross-reactivity)

Analytical specificity was evaluated with samples from clinical isolates, external quality assessment rounds, and commercial controls or panels.

Sixty-seven pathogens were analyzed, including bacteria, viruses, protozoa, and fungi clinically and biologically related to *S. agalactiae*. A minimum concentration of 1 × 10^6^ CFU/mL or 1 × 10^6^ copies/mL, as applicable, was used. Table S4 lists the pathogens used in the analytical specificity evaluation of the LDT-GBS assay, their sources, and their origin.

Bacterial suspensions from clinical isolates were prepared, and the turbidity was adjusted to the 0.5 McFarland standard with isotonic saline solution (BioMérieux SA). All bacterial suspensions were stabilized in the Aptima Specimen Transfer medium (Hologic) following the manufacturer’s specifications (55). All samples were tested in duplicate using the LDT-GBS assay on the Panther Fusion Open Access system (Hologic). *S. agalactiae* serotype Ia (ATCC 12386) was used as a positive control at a nominal concentration of 1 × 10^6^ CFU/mL.

### VIASURE *Streptococcus* B Real-Time comparative assay

All clinical samples enriched in Lim broth were subjected to external lysis and subsequent automated isolation of bacterial DNA using the MagNA Pure 24 Total DNA Isolation Kit (Roche Diagnostics, Indiana, USA) on the MagNA Pure 24 instrument (Roche Molecular Systems, New Jersey, USA). Briefly, 250 µL of enriched broth was added to a mixture consisting of 250 µL of Magna Pure Bacterial Lysis Buffer (Roche Diagnostics) and 50 µL of proteinase K (Roche Diagnostics, Mannheim, Germany). The resulting solution was incubated for 10 min at 56°C and 1,400 RPM and then for 10 min at 90°C and 1,400 RPM. Bacterial DNA extraction and isolation were performed following the manufacturer’s instructions: “Pathogen 1000 3.1” protocol, with a sample volume (lytic mixture) of 500 µL and an elution volume of 50 µL, was used.

The VIASURE *Streptococcus* B Real-Time amplification and detection reagent (CerTest Biotec) contains all specific (primers and fluorescent detection probes) and generic (reverse transcriptase, DNA polymerase, salts, amplification buffers, and other additives) components for the selective amplification of the target sequence of the *S. agalactiae cfb* gene (encoding the Christie-Atkins-Munch-Peterson factor) using qPCR. The kit is provided with ready-to-use lyophilized amplification components, rehydration buffer, PCR-grade water, and quality controls (positive and negative controls). This kit was used following the manufacturer’s instructions (56). Briefly, lyophilized amplification components were reconstituted with 15 µL of rehydration buffer and allowed to stand at room temperature (18°C–25°C) for 10 min. Then, 5 µL of eluate (template DNA) was added to complete the reaction volume (20 µL). Amplification and detection were performed on the CFX96-IVD analyzer (Bio-Rad) using the following protocol: one cycle for 2 min at 95°C and 45 cycles for 10 s at 95°C and 50 s at 60°C, with fluorescence readings at 60°C. The results were analyzed and interpreted according to the test manufacturer’s instructions.

### LDT-GBS and Panther Fusion GBS assays

Lim broth-enriched samples were stabilized in the Aptima Specimen Transfer medium (Hologic) following the manufacturer’s specifications (55). Stabilized samples were stored at 2°C–8°C for no more than 10 days, then analyzed using the test under evaluation, LDT-GBS, and the comparative test, Panther Fusion GBS (Hologic).

The Panther Fusion GBS amplification and detection reagent contains all specific (primers and fluorescent detection probes) and generic (reverse transcriptase, DNA polymerase, and salts) components for the selective amplification of the target sequences of the *S. agalactiae cfb* and *sip* genes. The kit is provided with ready-to-use lyophilized amplification components in sealed cartridges for up to 12 tests in an eight-cartridge box. The Panther Fusion instrument automates the reconstitution and preparation of the reaction mixture.

For the LDT-GBS assay, the PPR and Panther Fusion Open Access RNA/DNA Enzyme Cartridges were loaded into the corresponding compartments of the Fusion module. Panther Fusion GBS assay cartridges were loaded into the enzyme compartment. After loading the common and generic components of both assays and the samples and corresponding controls, the instrument user interface was used to run the assay. By default, bacterial DNA extraction and isolation start with the aspiration of 300 µL of the sample and end with the elution of the nucleic acids in a volume of 50 µL. Amplification and detection using qPCR were performed for both assays in the Fusion module in a reaction volume of 25 µL [20 µL of MMX (PPR + enzyme) and 5 µL of the eluate].

For the commercial Panther Fusion GBS assay, run validity criteria and parameters for the analysis and interpretation of results are unavailable to the end user, as the Panther Fusion system automatically assigns a result to the samples. For the LDT-GBS assay, a “positive” result was assigned when (i) the sigmoid fluorescence curve exceeded the predefined fluorescence threshold (1,000 RFU), and (ii) the *C*_*t*_ value was ≤39. A “negative” result was assigned when (i) the fluorescence curve did not exceed the predefined fluorescence threshold; (ii) the sigmoid fluorescence curve exceeded the defined threshold but with a *C*_*t*_ of >39; and (iii) there was a positive internal control. All samples with an “invalid” result [negative in both detection channels (GBS and IC-X)] were excluded from the analysis. Additional information on criteria for positivity, channel validity, and sample validity, as well as controls for the LDT-GBS assay in the Panther Fusion Open Access system, is provided in the supplemental archive, LDT-GBS myAccess Protocol.

### Isolation and identification through culture/mass spectrometry

The pathogens used in the analytical specificity evaluation were isolated from urine and perianal/vaginal swabs from routine laboratory samples, following standard procedures (57).

For GBS isolation, all clinical samples (rectovaginal swabs from pregnant women) previously enriched in Lim broth were plated on blood agar. For this, 50 µL of enriched broth was plated in a Petri dish containing the culture medium and was incubated at 36°C ± 1°C for 48 h in the presence of 5% CO_2_ under aerobic conditions. The cultures were examined for beta-hemolytic colonies suggestive of GBS.

All culture isolates were identified using MALDI-TOF mass spectrometry following the manufacturer’s instructions for the MALDI Biotyper Sirius instrument (Bruker, Massachusetts, USA). The BDAL library, v.12.0 (11 897 MSP, rev. 2023), and MBT Compass (RUO/GP) software, v.4.1.100, were used. Identification scores of ≥ 2.0 were considered acceptable.

### Agreement assessment between qPCR assays

Using 2 × 2 contingency tables, positive percent agreement (PPA), negative percent agreement (NPA), OPA, and Cohen’s kappa coefficient (*k*) were calculated as estimators of the agreement between the results of the LDT-GBS assay and the results of the comparative commercial qPCR-based assays: Panther Fusion GBS (Hologic) and VIASURE *Streptococcus* B Real-Time (CerTest Biotec). Samples with discordant results were retested using all qPCR assays. Consistently discordant samples were submitted to consensus.

An OPA ≥90% and a *k* > 0.8 were considered indicators of excellent (acceptable) agreement (58).

### Diagnostic performance

The diagnostic sensitivity and specificity, as well as the positive predictive value (PPV) and negative predictive value (NPV) of the LDT-GBS assay, culture, and comparative qPCR-based assays—Panther Fusion GBS (Hologic) and VIASURE *Streptococcus* B Real-Time (CerTest Biotec)—were calculated by comparison with the expected results. A consensus of results from all assays included in the evaluation—LDT-GBS, culture, and comparative commercial qPCR-based assays—was assumed to be an expected result. Consensus was defined by the following rules: (A) a positive result if (i) the reference method (culture) yielded a positive result, regardless of the result of the qPCR assays, and (ii) at least two qPCR assays yielded a positive result, regardless of the result of the reference method; (B) a negative result if (i) all assays yielded a negative result and (ii) only one qPCR assay yielded a positive result. The prevalence of GBS colonization was calculated considering two scenarios: exclusively by culture and by consensus. For the above, the following equations were used:


(ii) Sensitivity(%)=TP/(TP+FN)×100%

(iii) Specificity(%)=TN/(FP+TN)×100%

(iv) PPV(%)=TP/(TP+FP)×100%

(v) NPV(%)=TN/(FN+TN)×100%

(vi) Prevalence(%)=(TP+FN)/n×100%

(vii) PR(%)=P/n×100%

where TP represents the true positives; FP represents the false positives; FN represents the false negatives; TN represents the true negatives; *n* represents the total number of samples; PR represents the positive rate; and *P* represents the number of positive results detected by the assay.

Optimal diagnostic sensitivity and specificity for screening were defined as any value ≥90% (threshold or cutoff value) (11).

### Statistical analysis

The Wilson score method for binomial proportions (59) was used to calculate the 95% CIs of percent agreements (PPA, NPA, and OPA), PR, prevalence, and diagnostic performance parameters (sensitivity, specificity, PPV, and NPV). The 95% CI of Cohen’s kappa coefficient was calculated using the Wald method for binomial proportions (59).

The nonparametric McNemar-Mosteller test was used to evaluate the statistical significance of the differences between PRs and the diagnostic sensitivity and specificity of the assays in the present study—culture, LDT-GBS, and comparative commercial qPCR-based assays—and of the difference between the prevalence calculated with respect to the culture and with respect to the consensus. The statistical significance of the difference between the prevalence estimated considering the consensus and the expected prevalence (44%) (21) was analyzed using Pearson’s chi-square test.

Statistical analyses were performed using Analyse-it software for Microsoft Excel, Ultimate Edition, v.6.15.4 (Analyse-it Software Ltd., Leeds, UK). The statistical significance level was set at 5% (*P* < 0.05).

## SUPPLEMENTAL MATERIAL

LDT-GBS myAccess Protocol spectrum.00057-24-s0001.pdf LDT-GBS protocol developed using MyAccess software (Hologic) for Panther Fusion Open Access{trade mark, serif} system (Hologic).

Supplemental material spectrum.00057-24-s0002.docx Fig. S1 to S3; Tables S1 to S4.

ASM does not own the copyrights to Supplemental Material that may be linked to, or accessed through, an article. The authors have granted ASM a non-exclusive, world-wide license to publish the Supplemental Material files. Please contact the corresponding author directly for reuse.
