# Supplementary material for: Development and performance evaluation of a qPCR-based assay for the fully automated detection of group B Streptococcus (GBS) on the Panther Fusion Open Access system
Source: Microbiol Spectr. 2024 Apr 29;12(6):e00057-24. doi: 10.1128/spectrum.00057-24 (PMC11237499; doi:10.1128/spectrum.00057-24)
Supplement: LDT-GBS myAccess Protocol — LDT-GBS protocol developed using MyAccess software (Hologic) for Panther Fusion Open Access{trade mark, serif} system (Hologic). [file spectrum.00057-24-s0001.pdf]

Protocol Definition: LDT-GBS (Version 4.0.8.0)

Protocol Identification

**Protocol Name**	LDT-GBS
**Version**	4.0.8.0
**Extraction Type**	Low - Viral/Bacterial (1.50)
**Results Processing**	Qualitative
**Author**	Andy Caballero
**Created On**	12/11/2023 3:40:17 PM
**Modified On**	--
**Exported On**	12/11/2023 3:43:01 PM
**Description**	S. agalactiae detection: sip gene.

Extraction & PCR Details

**Sample Tube Cap Type**	Hologic Pierceable Cap
**Sample Aspiration Height**	Low
**Template**	DNA
**Template Volume**	5 µL
**Extraction Volume**	300 µL
**Reagent Kit**	FCR-X/FER-X
**Elution Buffer Volume**	50 µL
**Enzyme Cartridge Type**	RNA/DNA Enzyme

Targets Setup

Selected	Channel	Example Dye	Excitation/Detection Wavelength (nm)	Analyte Name	Additional Information (Optional)
Yes	1	FAM	460/517	GBS	S. agalactiae sip gene
No	2	HEX	532/563	--	--
No	3	ROX	586/625	--	--
No	4	Quasar 670	625/660	--	--
Yes	5	Quasar 705	690/720	IC	Hologic DNA IC-X

Protocol Definition: LDT-GBS (Version 4.0.8.0)

Thermocycler Setup

Profile: DNA

Projected Thermocycler Runtime (hh:mm:ss): 00:50:53

Stage Name 1: Holding Stage
Number Of Cycles 1

Step Name Step 1
Temperature (°C) 95
Duration (mm:ss) 02:00
Optics On No

Stage Name 2: Cycling Stage
Number Of Cycles 45

Step Name Step 1
Temperature (°C) 95
Duration (mm:ss) 00:08
Optics On No

Step Name Step 2
Temperature (°C) 60
Duration (mm:ss) 00:25
Optics On Yes

Parameters

Curve Correction Method Option 2
Enhanced Resolution Enabled Yes

Curve Correction

Analyte Name	Channel	Analysis Start Cycle	Baseline Correction	
			Enable	Slope Limit
GBS	1-FAM	10	Yes	50
IC	5-Quasar 705	10	Yes	50

Crosstalk Correction

Analyte Name	Emit\Receive	1	5
GBS	**1-FAM**	--	0.00 %
IC	**5-Quasar 705**	0.00 %	--

Protocol Definition: LDT-GBS (Version 4.0.8.0)

Positivity Criteria

Analyte Name	Channel	Ct Threshold	Minimum Slope at Threshold		Maximum Ct	
			Enable	Value	Enable	Value
GBS	1-FAM	1000	Yes	220	Yes	39
IC	5-Quasar 705	1000	Yes	350	Yes	36

Channel Validity Criteria

Analyte Name	Channel	Minimum Background Fluorescence		Maximum Background Fluorescence		Lowest Valid Ct	
		Enable	Value	Enable	Value	Enable	Value
GBS	1-FAM	Yes	2000	Yes	6000	Yes	12
IC	5-Quasar 705	Yes	400	Yes	2000	Yes	25

Sample Validity Criteria

Minimum of 1 positive channel required Yes
Internal Control (IC) in Channel 5-Quasar 705

Controls

Control Failure Handling Invalidate Specimens
Control Set Expiration 1 days

Control Name	Control Type	Analyte Name	Channel	Minimum Ct Value	Maximum Ct Value
GBS NC	Negative	IC	5-Quasar 705	25	36
GBS PC	Positive	GBS	1-FAM	12	39

Export Settings

Protocol Lock Status Off
Sample Results to LIS Mode --
Revision Comments Slope at Threshold and min and max RFU Backgound was ajusted.

Notes

--
