# Supplementary material for: Development and performance evaluation of a qPCR-based assay for the fully automated detection of group B Streptococcus (GBS) on the Panther Fusion Open Access system
Source: Microbiol Spectr. 2024 Apr 29;12(6):e00057-24. doi: 10.1128/spectrum.00057-24 (PMC11237499; doi:10.1128/spectrum.00057-24)
Supplement: Supplemental material — Fig. S1 to S3; Tables S1 to S4. [file spectrum.00057-24-s0002.docx]

Development and Performance Evaluation of a qPCR-Based Assay for the Fully Automated Detection of Group B *Streptococcus* (GBS) on the Panther Fusion® Open Access™ System

Andy Caballero Méndez,^a^# Roberto A. Reynoso de la Rosa,^a^ Miguel E. Abreu Bencosme,^a^ Mayeline N. Sosa Ortiz,^a^ Eliezel Pichardo Beltré,^a^ Darah M. de la Cruz García,^a^* Nelson J. Piñero Santana,^a^ Joana C. Bacalhau de León^b^

^a^Molecular Biology Department, Referencia Laboratorio Clínico, Zona Industrial de Herrera, Santo Domingo Oeste, Santo Domingo, Dominican Republic

^b^Microbiology Department, Referencia Laboratorio Clínico, Zona Industrial de Herrera, Santo Domingo Oeste, Santo Domingo, Dominican Republic

Running Head: GBS Antepartum Screening via Panther Fusion® LDT-qPCR

#Address correspondence to Andy Caballero Méndez, andyc@labreferencia.com.

*Present address: Worcester Polytechnic Institute, Worcester, Massachusetts, United States of America

# Supplemental Tables

**Table S1.** Diagnostic sensitivity and specificity of qPCR-based assays vs a consensus previously reported (1-8) and intralaboratory assessed: antepartum GBS screening.

| **Assay (Author)** | **Manufacturer** | **Target** | **Contingency table** | | | | **N** | **Sensitivity** | | | **Specificity** | | |
| --- | --- | --- | --- | --- | --- | --- | --- | --- | --- | --- | --- | --- | --- |
|  |  |  | **TP** | **FP** | **FN** | **TN** |  | **%** | **95 %IC** | | **%** | **95 %IC** | |
| *Previously reported* | | | | | | | | | | | | | |
| Panther Fusion GBS (Hologic) (Shin et al., 2019) | Hologic | cfb / sip | 141 | 2 | 6 | 351 | 500 | 95.9 | (91.4 | to 98.1) | 99.4 | (98.0 | to 99.8) |
| LC StrepB (Roche Diagnostics) (Goodrich et al., 2007) | Roche Diagnostics |  | 59 | 0 | 1 | 140 | 200 | 98.3 | (91.1 | to 99.7) | 100.0 | (97.3 | to 100.0) |
| GeneOhm StrepB (BD) (Goodrich et al., 2007) | BD | cfb | 56 | 4 | 4 | 136 | 200 | 93.3 | (84.1 | to 97.4) | 97.1 | (92.9 | to 98.9) |
| AmpliVue GBS (Quidel) (Miller et al., 2015) | Quidel | atoB (Tiolasa) | 54 | 6 | 2 | 138 | 200 | 96.4 | (87.9 | to 99.0) | 95.8 | (91.2 | to 98.1) |
| BD MAX GBS (BD) (Miller et al., 2015) | BD | cfb | 56 | 6 | 0 | 138 | 200 | 100.0 | (93.6 | to 100.0) | 95.8 | (91.2 | to 98.1) |
| BD MAX GBS (BD) (Bogiel et al., 2021) | BD | cfb | 52 | 6 | 0 | 192 | 250 | 100.0 | (93.1 | to 100.0) | 97.0 | (93.5 | to 98.6) |
| ARIES GBS (Luminex - DiaSorin) (Shin et al., 2019) | Luminex - DiaSorin | cfb | 142 | 13 | 5 | 340 | 500 | 96.6 | (92.3 | to 98.5) | 96.3 | (93.8 | to 97.8) |
| Xpert GBS LB (Cepheid) (Shin et al., 2019) | Cepheid | cfb | 141 | 6 | 6 | 347 | 500 | 95.9 | (91.4 | to 98.1) | 98.3 | (96.3 | to 99.2) |
| Xpert GBS LB XC (Cepheid) (Thwe et al., 2022) | Cepheid | GTFP / LysR | 142 | 6 | 1 | 472 | 621 | 99.3 | (96.1 | to 99.9) | 98.7 | (97.3 | to 99.4) |
| Strep B Real-TM (Sacace) (Peris et al., 2022) | Sacace |  | 115 | 0 | 0 | 298 | 413 | 100.0 | (96.8 | to 100.0) | 100.0 | (98.7 | to 100.0) |
| VIASURE GBS (CerTest) (Peris et al., 2022) | CerTest | cfb | 112 | 0 | 3 | 298 | 413 | 97.4 | (92.6 | to 99.1) | 100.0 | (98.7 | to 100.0) |
| LDT PCR-A (sip) (Carrillo-Ávila et al., 2018) | LDT | *sip* | 86 | 0 | 3 | 231 | 320 | 96.6 | (90.6 | to 98.8) | 100.0 | (98.4 | to 100.0) |
| LDT PCR-B (cfb) (Carrillo-Ávila et al., 2018) | LDT | *cfb* | 85 | 2 | 4 | 229 | 320 | 95.5 | (89.0 | to 98.2) | 99.1 | (96.9 | to 99.8) |
| *Intralaboratory assessed* | | | | | | | | | | | | | |
| Panther Fusion GBS (Hologic) | Hologic | cfb / sip | 81 | 0 | 1 | 218 | 300 | 98.8 | (93.4 | to 99.8) | 100.0 | (98.3 | to 100.0) |
| VIASURE GBS (CerTest) | CerTest | cfb | 82 | 1 | 0 | 217 | 300 | 100.0 | (95.5 | to 100.0) | 99.5 | (97.4 | to 99.9) |
| LDT-GBS | LDT | sip | 82 | 0 | 0 | 218 | 300 | 100.0 | (95.5 | to 100.0) | 100.0 | (98.3 | to 100.0) |

*TP, true positive; FN, false negative; FP, false positive; TN, true negative; N, total number of samples; 95%CI, 95% confidence interval.*

**Table S2.** List of representative isolates/strains of the *S. agalactiae* serotypes used in *sip* gene alignment for oligo designing.

| **Source** | **Source ID / Accession Number** | **Loci** | **Serotype** | **Isolate / Strain** | **Country** |
| --- | --- | --- | --- | --- | --- |
| PubMLST | 1048 | SAG0032 | Ia | ERR1624733 | The Netherlands |
| PubMLST | 1062 | SAG0032 | Ib | ERR1624735 | The Netherlands |
| PubMLST | 1176 | SAG0032 | II | ATCC 13813 | Unknown |
| PubMLST | 21728 | SAG0032 | III | PHEGBS0524 | UK |
| PubMLST | 1130 | SAG0032 | IV | ERR1624742 | The Netherlands |
| PubMLST | 1555 | SAG0032 | V | SS100 | USA |
| PubMLST | 3906 | SAG0032 | VI | ERR1659756 | The Netherlands |
| PubMLST | 5605 | SAG0032 | VII | KMB-572 | Slovakia |
| PubMLST | 21207 | SAG0032 | VIII | PHEGBS0003 | UK |
| PubMLST | 5906 | SAG0032 | IX | WOL-1-75 | Australia |
| Genbank | CP019818.1 | N/A | Ib | SA184 | Brazil |
| PubMLST | 1420 | SAG0032 | Ib | SS1219 | Unknown |

**Table S3.** Microorganisms whose sequences were employed for the *in silico* cross-reactivity (specificity) evaluation of the primers and probe used in the LDT-GBS assay.

| **Microorganism (taxid)** | **Microorganism (taxid)** |
| --- | --- |
| *Bacillus cereus* (taxid: 1396) | *Streptococcus pyogenes* (taxid: 1314) |
| *Yersinia enterocolitica* (taxid: 630) | *Streptococcus ratti* (taxid: 1341) |
| *Anaerococcus prevotii* (taxid: 33034) | *Staphylococcus lugdunensis* (taxid: 28035) |
| *Propionibacterium acnes* (taxid: 1747) | *Acinetobacter baumannii* (taxid: 470) |
| *Clostridium difficile* (taxid: 1496) | *Staphylococcus aureus* (taxid: 1280) |
| *Fusobacterium nucleatum* (taxid: 851) | *Staphylococcus epidermidis* (taxid: 1282) |
| *Bifidobacterium adolescentis* (taxid: 1680) | *Shigella sonnei* (taxid: 624) |
| *Candida albicans* (taxid: 5476) | *Citrobacter freundii* (taxid: 546) |
| *Candida glabrata* (taxid: 5478) | *Enterococcus gallinarum* (taxid: 1353) |
| *Candida tropicalis* (taxid: 5482) | *Acinetobacter lwoffii* (taxid: 28090) |
| *Cryptococcus neoformans* (taxid: 5207) | *Pseudomonas aeruginosa* (taxid: 287) |
| *Klebsiella pneumoniae* (taxid: 573) | *Streptococcus ricetid* (taxid: 1333) |
| *Proteus mirabilis* (taxid: 584) | *Haemophilus influenzae* (taxid: 727) |
| *Alcaligenes faecalis* (taxid: 511) | *Klebsiella oxytoca* (taxid: 571) |
| *Enterobacter aerogenes* (taxid: 548) | *Streptococcus bovis* (taxid: 1335) |
| *Stenotrophomonas maltophilia* (taxid: 40324) | *Streptococcus parasanguinis* (taxid: 1318) |
| *Campylobacter jejuni* (taxid: 197) | *Streptococcus equi* (taxid: 1336) |
| *Providencia stuartii* (taxid: 588) | *Enterococcus durans* (taxid: 53345) |
| *Micrococcus luteus* (taxid: 1270) | *Lactobacillus plantarum* (taxid: 1590) |
| *Staphylococcus haemolyticus* (taxid: 1283) | *Streptococcus dysgalactiae* (taxid: 1334) |
| *Enterococcus faecalis* (taxid: 1351) | *Streptococcus constellatus* (taxid: 76860) |
| *Pseudomonas fluorescens* (taxid: 294) | *Streptococcus oralis* (taxid: 1303) |
| *Staphylococcus saprophyticus* (taxid: 29385) | *Bacillus coagulans* (taxid: 1398) |
| *Proteus vulgaris* (taxid: 585) | *Streptococcus pseudoporcinus* (taxid: 361101) |
| *Toxoplasma gondii* (taxid: 5811) | *Streptococcus mitis* (taxid: 28037) |
| *Enterococcus faecium* (taxid: 1352) | *Finegoldia magna* (taxid: 1260) |
| *Escherichia coli* (taxid: 562) | *Peptostreptococcus anaerobius* (taxid: 1261) |
| *Streptococcus anginosus* (taxid: 1328) | *Anaerococcus lactolyticus* (taxid: 33032) |
| *Prevotella oralis* (taxid: 28134) | Epstein‒Barr-virus EBV (taxid: 10376) |
| *Streptococcus canis* (taxid: 1329) | *Bacteroides fragilis* (taxid: 817) |
| *Lactobacillus delbrueckii* (subsp. Lactis) (taxid: 29397) | *Bordetella pertussis* (taxid: 520) |
| *Corynebacterium* sp. (taxid: 1720) | *Chlamydia trachomatis* (taxid: 813) |
| *Neisseria gonorrhoeae* (taxid: 485) | CMV (taxid: 12305) |
| *Streptococcus pneumoniae* (taxid: 1313) | *Hafnia alvei* (taxid: 569) |
| *Streptococcus mutans* (taxid: 1309) | *Trichomonas vaginalis* (taxid: 5722) |
| *Corynebacterium urealyticum* (taxid: 43771) | HIV-1 (taxid: 11676) |
| *Lactobacillus reuteri* (taxid: 1598) | *Moraxella catarrhalis* (taxid: 480) |
| uncultured *Lactobacillus* sp. (taxid: 153152) | *Mycoplasma genitalium* (taxid: 2097) |
| *Lactobacillus casei* (taxid: 1582) | *Prevotella melaninogenica* (taxid: 28132) |
| *Lactobacillus acidophilus* (taxid: 1579) | Rubella virus (taxid: 11041) |
| *Streptococcus gordonii* (taxid: 1302) | *Serratia marcescens* (taxid: 615) |
| *Enterobacter cloacae* (taxid: 550) | *Streptococcus intermedius* (taxid: 1338) |
| *Aeromonas hydrophila* (taxid: 644) | HPV16 (taxid: 333760) |
| *Moraxella atlantae* (taxid: 34059) | HBV (taxid: 10407) |
| *Prevotella bivia* (taxid: 28125) | HCV (taxid: 3052230) |
| *Pasteurella aerogenes* (taxid: 749) | HSV-1 (taxid: 10298) |
| *Rhodococcus equi* (taxid: 43767) | HSV2 (taxid: 10310) |
| *Listeria monocytogenes* (taxid: 1639) | VZV (taxid: 10335) |
| *Lactobacillus gasseri* (taxid: 1596) | *Arcanobacterium pyogenes* (taxid: 1661) |
| *Peptoniphilus asaccharolyticus* (taxid: 1258) | *Mobiluncus curtisii* (taxid: 2051) |
| *Atopobium vaginae* (taxid: 82135) | *Gardnerella vaginalis* (taxid: 2702) |
| *Morganella morganii* (taxid: 582) | *Salmonella* sp. (taxid: 590) |
| *Shigella flexneri* (taxid: 623) | *Streptococcus acidominimus* (taxid: 1326) |
| *Anaerococcus tetradius* (taxid: 33036) | JC virus × BK virus (taxid: 178534) |

**Table S4.** Pathogens used in the evaluation of the analytical specificity (cross-reactivity) of the LDT-GBS assay.

| **Sample content** | **Source** | **Manufacturer** |  | **Sample content** | **Source** | **Manufacturer** |
| --- | --- | --- | --- | --- | --- | --- |
| *S. agalactiae* | QC | ATCC |  | *P. vulgaris* | CI | – |
| *S. pseudoporcinus* | CI | – |  | *C. koseri* | CI | – |
| *S. anginosus* | CI | – |  | *M. morganii* | CI | – |
| *S. pyogenes* | CI | – |  | *E. cloacae* | CI | – |
| *S. pneumoniae* | CI | – |  | *A. caviae* | CI | – |
| *S. oralis* | CI | – |  | *P. putida/monteilii* | CI | – |
| *S. constellatus* | CI | – |  | *E. coli* | CI | – |
| *S. gallolyticus* | CI | – |  | *E. faecalis* | CI | – |
| *S. canis* | CI | – |  | *S. lugdunensis* | CI | – |
| *S. salivarius* | CI | – |  | *K. pneumoniae* | CI | – |
| *T. vaginalis* | CP | Qnostics |  | *P. mirabilis* | CI | – |
| *M. genitalium* | CP | Qnostics |  | *S. aureus* | CI | – |
| *U. urealyticum* | CP | Qnostics |  | *P. aeruginosa* | CI | – |
| *M. hominins* | CP | Qnostics |  | *S. saprophyticus* | CI | – |
| *G. vaginalis* | CP | Qnostics |  | *V. Streptococcus* | EQAS | CAP |
| *N. gonorrhoeae* | EQAS | CAP |  | Rotavirus | CP | Qnostics |
| *C. trachomatis LGV* | EQAS | CAP |  | Astrovirus | CP | Qnostics |
| *C. trachomatis SW* | CP | Qnostics |  | Sapovirus | CP | Qnostics |
| *T. pallidum* | EQAS | QCMD |  | HBV | CP | Seracare |
| *C. jejuni* | CP | Qnostics |  | HCV | CP | Seracare |
| *P. shigelloides* | CP | Qnostics |  | HIV-1 | CP | Seracare |
| *C. lari* | CP | Qnostics |  | HSV-1 (HHV1) | QC | Qnostics |
| *C. difficile* | CP | Qnostics |  | HSV-2 (HHV2) | QC | Qnostics |
| *S. enteritidis* | CP | Qnostics |  | VZV (HHV3) | QC | Qnostics |
| *S. flexneri* | CP | Qnostics |  | EBV (HHV4) | QC | Qnostics |
| *Y. enterocolitica* | CP | Qnostics |  | CMV (HHV5) | EQAS | CAP |
| *G. intestinalis* | CP | Qnostics |  | HHV6 | QC | Qnostics |
| *C. parvum* | CP | Qnostics |  | Norovirus GI | CP | Qnostics |
| *E. histolytica* | CP | Qnostics |  | Norovirus GII | CP | Qnostics |
| *S. epidermidis* | EQAS | CAP |  | Adenovirus 41 | CP | Qnostics |
| *G. vaginalis + A. vaginae* | CI | – |  | HPV18 | CP | Qnostics |
| *Lactobacillus* spp. | EQAS | CAP |  | *C. albicans* | CP | Qnostics |
| *K. variicola* | CI | – |  | *C. krusei* | CP | Qnostics |
| *A. baumannii* | CI | – |  | *C. glabrata* | CP | Qnostics |

*CAP, College of American Pathologists; EQA, external quality assessment; ATCC, American Type Culture Collection; QCMD, Quality Control for Molecular Diagnostics; QC, quality control; CI, clinical isolate; CP, commercial panel; EQAS, external quality assessment sample; HBV, Hepatitis B Virus; HCV, Hepatitis C Virus; HIV-1, Human immunodeficiency virus type 1; HSV, Herpes simplex virus type 1 (HSV-1) and 2 (HSV-2); VZV, Varicella-zoster virus; CMV, Cytomegalovirus; HHV, Human Herpes Virus type 1 to 6 (HHV1 – HHV6); HPV18, Human papillomavirus type 18.*

# Supplemental Figures


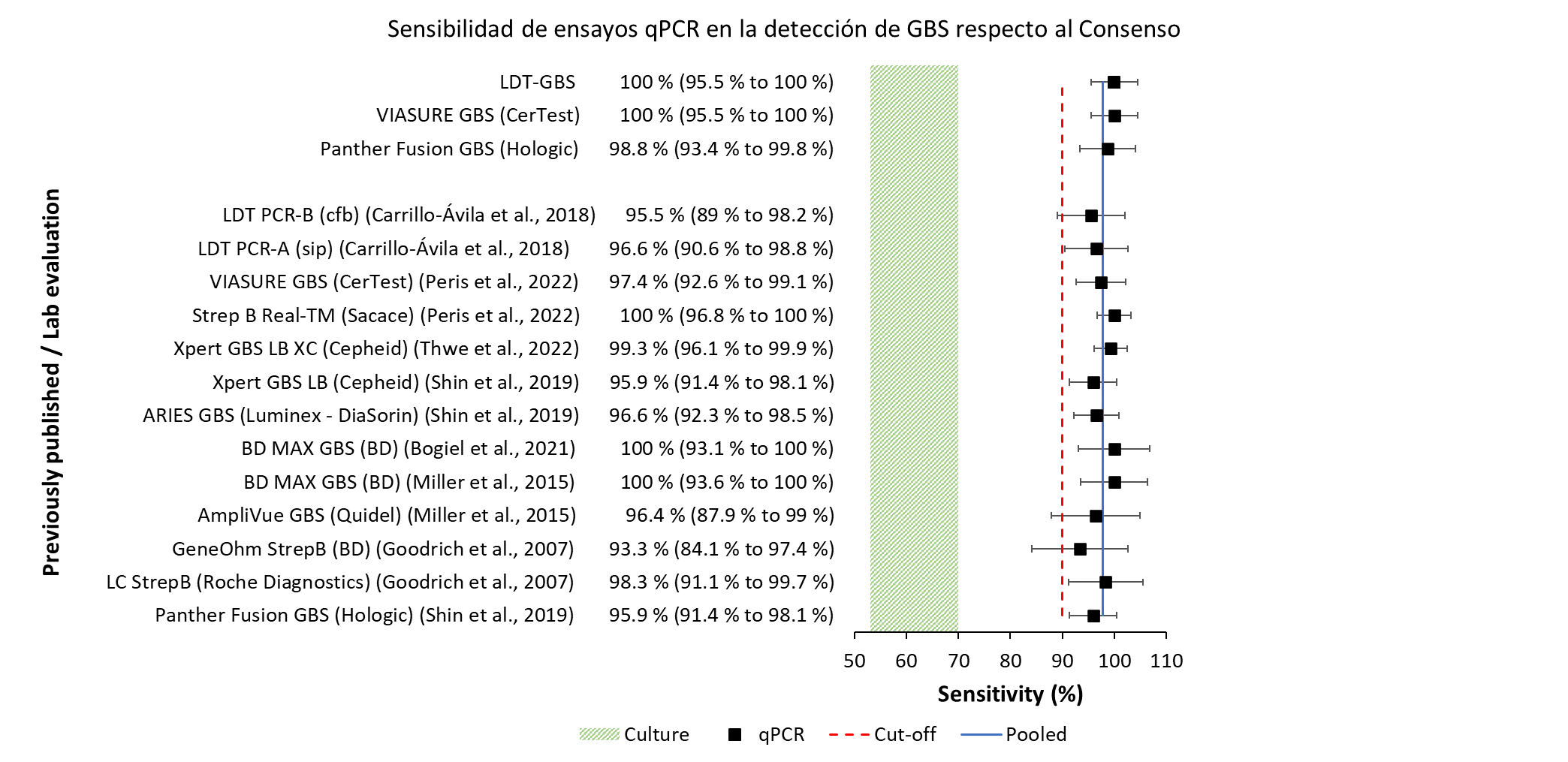


**Fig. S1.** Diagnostic sensitivity of qPCR-based assays vs a consensus previously reported (1-8) and intralaboratory assessed: antepartum GBS screening. Green rectangle: culture sensitivity (53–70%). Red dashed line: US CDC cutoff (90%) (9). Solid blue line: pooled sensitivity.


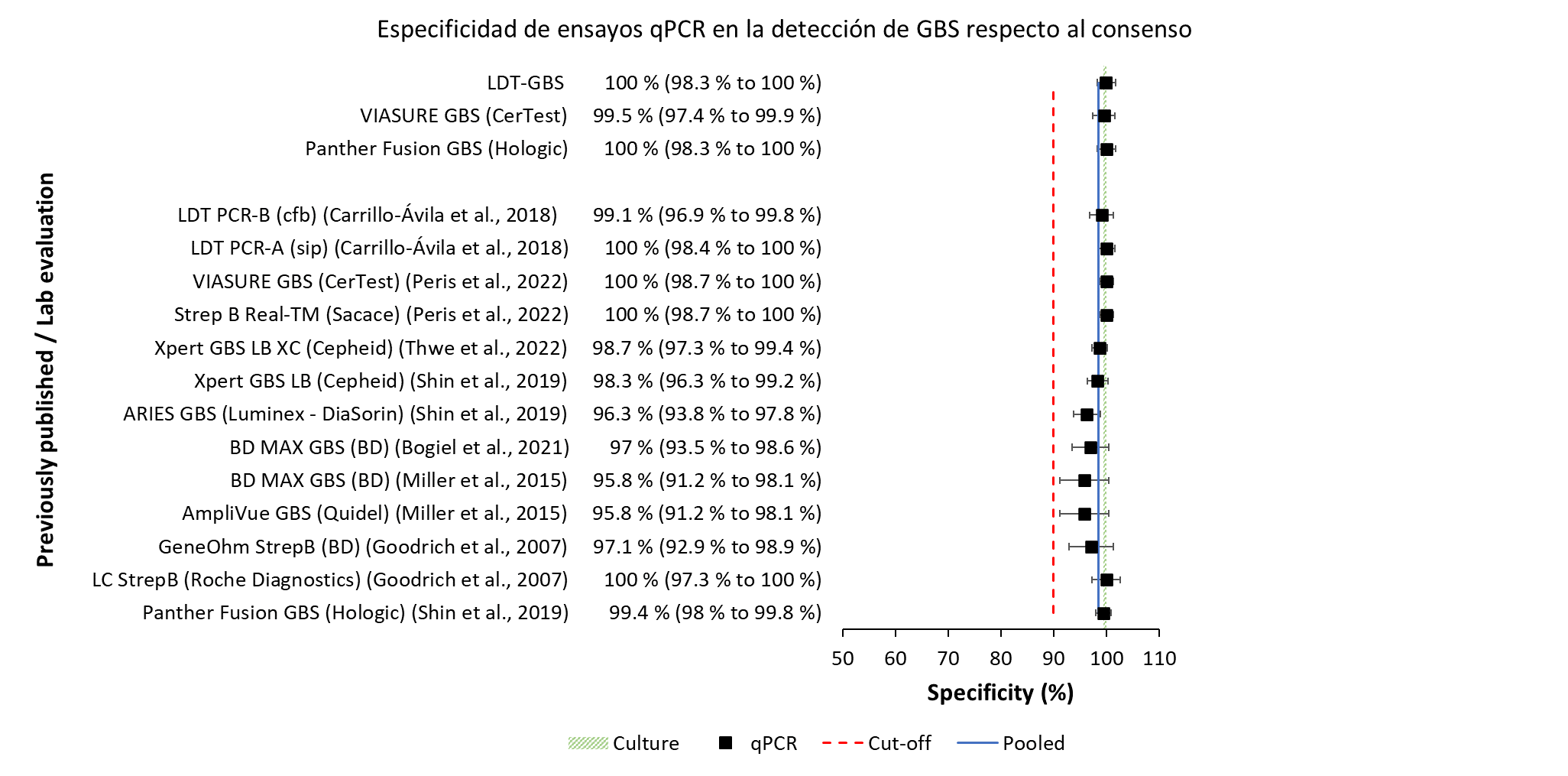


**Fig. S2.** Diagnostic specificity of qPCR-based assays vs a consensus previously reported (1-8) and intralaboratory assessed: antepartum GBS screening. Green dashed line: culture specificity (100%). Red dashed line: US CDC cutoff (90%) (9). Solid blue line: pooled specificity.

**Fig. S3.** Graphical Ct value representation of the analytical sensitivity evaluation results. The Ct values of the positive sample replicates of the six levels of the LoD dilution panel versus the nominal CFU/mL are presented. Solid line: LoD (C_95_) in terms of the Ct value. Dotted-dashed line: concentration of the analyte corresponding to a probability of success of 50% (C_50_). Dotted line: Maximum of all Ct values (Max Ct value). Dashed line: Cutoff calculated. The Ct equivalent values of C_95_ and C_50_ were estimated using the graphical equation of the amplification efficiency experiment (data not shown).

# References

1. Shin JH, Pride DT. 2019. Comparison of Three Nucleic Acid Amplification Tests and Culture for Detection of Group B Streptococcus from Enrichment Broth. J Clin Microbiol 57:1-9.

2. Goodrich JS, Miller MB. 2007. Comparison of culture and 2 real-time polymerase chain reaction assays to detect group B Streptococcus during antepartum screening. Diagn Microbiol Infect Dis 59:17-22.

3. Mueller M, Henle A, Droz S, Kind AB, Rohner S, Baumann M, Surbek D. 2014. Intrapartum detection of Group B streptococci colonization by rapid PCR-test on labor ward. Eur J Obstet Gynecol Reprod Biol 176:137-141.

4. Miller SA, Deak E, Humphries R. 2015. Comparison of the AmpliVue, BD Max System, and illumigene Molecular Assays for Detection of Group B Streptococcus in Antenatal Screening Specimens. J Clin Microbiol 53:1938-1941.

5. Bogiel T, Depka D, Zalas-Więcek P, Rzepka M, Kruszyńska E, Gospodarek-Komkowska E. 2021. Application of the appropriate molecular biology-based method significantly increases the sensitivity of group B streptococcus detection results. J Hosp Infect 112:21-26.

6. Thwe PM, Faron ML, Pride DT, Cruz A, Gerstbrein D, Nahmod KA, Bigham L, Du X, Lu X, Moya S, Ren P. 2022. Multicenter Evaluation of the Cepheid Xpert GBS LB XC Test. J Clin Microbiol 60:1-6.

7. Peris MP, Martín-Saco G, Alonso-Ezcurra H, Escolar-Miñana C, Rezusta A, Acero R, Milagro-Beamonte A. 2022. Retrospective Study for the Clinical Evaluation of a Real-Time PCR Assay with Lyophilized and Ready-to-Use Reagents for Streptococcus agalactiae Detection in Prenatal Screening Specimens. Diagnostics 12:1-9.

8. Carrillo-Ávila JA, Gutiérrez-Fernández J, González-Espín AI, García-Triviño E, Giménez-Lirola LG. 2018. Comparison of qPCR and culture methods for group B Streptococcus colonization detection in pregnant women: evaluation of a new qPCR assay. BMC Infect Dis 18:1-8.

9. Filkins L, Hauser J, Robinson-Dunn B, Tibbetts R, Boyanton B, Revell P. 2020. Updated: July 23, 2021. Initially posted: March 10. Guidelines for detection and identification of group B Streptococcus, vol Updated: July 23, 2021. Initially posted: March 10. American Society for Microbiology, Washington, DC.
